# Research of the Dispersity of the Functional Sericite/Methylphenyl- Silicone Resin

**DOI:** 10.1371/journal.pone.0127735

**Published:** 2015-06-10

**Authors:** B. Jiang, C. C. Zhu, Y. D. Huang

**Affiliations:** 1 State Key Laboratory of Urban Water Resource and Environment, Harbin Institute of Technology, Harbin 150090; 2 Polymer Materials and Engineering Department, School of Chemical Engineering and Technology, Harbin Institute of Technology, P.O. Box: 1254, Harbin 150001, People’s Republic of China; Bascom Palmer Eye Institute, University of Miami School of Medicine, UNITED STATES

## Abstract

In order to improve the homogeneity and dispersity of the sericite in methylphenyl-silicone resin, the agglomerate state of the sericites was controlled effectively. The dispersive model of the sericite in methylphenyl-silicone resin was designed also. First, the modified sericite was prepared using hexadecyl trimethyl ammonium bromide as the intercalating agent. Then, functional sericite was incorporated into methylphenyl-silicone by terminal hydroxyl. The structure and dispersive performance of the hybrid polymers was charactered by analytical instruments. Scanning electron microscopy and Transmission electron microscope, Laser scanning confocal microscope and X-ray diffraction analysis showed that functional sericite was dispersed homogeneously in methylphenyl-silicone resin matrix. X-ray photoelectron spectroscopy analysis showed that the absorption peaks of the Si-OH band of methylphenyl-silicone resin were decreased and the Si-O-Si band was increased. This change evidently showed a significant role to enhance the reaction degree of the functional sericite in methylphenyl-silicone resin.

## Introduction

In the current study, nanometre-scale, self-assembly [1,2] and surface modified inorganic particles are [3] used frequently. Dispersity of the inorganic nano-particle is important problem [4,5,6]. Sericite is a layered silicate structure and has the perfect cleavage, flexibility, elasticity, low thermal conductivity, infusibility and high dielectric strength, it has been widely used in industry [7,8]. On the other hand, the sericite is applied as a kind of filler in the industry also, such as pigment, rubber, paper, and plastics, so on. The layered structure of the sericite is close-knit and not exchangeable ions in layers. The high bond energy is provided from the sericite structure. So, the interlayer structure of sericite was activated by the physical or chemistry method, and then activated sericite was expanded through intercalation polymerization.

Silicone resin matrix composites, as a kind of heat-resistant material, have many excellent dielectric properties, low heat expansion coefficient and light weight, so that silicone resin is a promising coating material, it has widely applied on electrical equipment, planes and vehicle so on [9]. The synthesis, modified method of the silicone resin as high performances composites is studied, such as nano-particle [10,11] and fiber reinforced [12,13], stem grafting [14], polymerize [15], crosslinking [16]. Especially, nano- particle reinforces silicone resin composites.

The chemical formula of the sericite is KAl_2_[AlSi_3_O_10_](OH)_2,_ Si-O tetrahedron structure of the sericite and Si-O backbone structure of the molecular of the silicone resin is similar. If the dispersion of sericite in the silicone resin can improve, the heat stability of the silicone resin can enhance. The aim of this study is develop a dispersive strategy of sericite in methylphenyl-silicone resin and designed the dispersive model.

## Experimental Section

### 1. Syntheses of the modified sericite/ methylphenyl-silicone resins nanocomposites.

The sericites and methylphenyl-silicone resin were obtained from Chuzhou and Shanghai Hersbit Chemical Co., Ltd, China, respectively. The crude sericites were exfoliated using chemical method. 1 g sericites were added gradually to mixed solution (20 mL H_2_SO_4_ (98%), 0.5 g NaNO_3_ and 3 g of KMnO_4_) at ice-bath, the temperature of the mixture was maintained below 20°C. The mixture was then stirred at 35°C for 120 min, 50 mL distilled water was slowly added, the temperature of the reaction solution was rises to 98°C for 30 min. The reaction was terminated by 100 mL distilled water followed by 1 mL H_2_O_2_ solution. The sericites were separated by centrifugation and washed by 10 ml 10% HCl solution, and then the sericites were collected using freezing dry for 24 h.

Sericites, N,N-Dimethyl formamide solution (concentration 5%) were loaded into a glass container. The mixture was sonicated for 6 h at room temperature. The solution was separated in a centrifuge giving rise to functional sericites sheets. Then, the sericites sheets were added the saturated NaCI solution (concentration 10%) by ion exchange. It was separated by centrifugation, washed repeatedly by deionized water until chlorides could not be detected. Finally, the product was obtained by freezing dry for 24 h.

Active surface reagent- hexadecyl trimethyl ammonium bromide (CTAB, 10 g) was added to 100 mL butyl alcohol, 1 g sericites were added gradually. The mixture was then stirred at 90°C for 8 h. The modified sericites were separated by centrifugation, and then above product was dried in a vacuum oven at 60°C.

The modified sericite and methylphenyl-silicone resin was mixed by the ultrasonic at -10°C-5°C ranges 2 h. The doping content of the modified sericite was 2% weight percent in methylphenyl-silicone matrix. Above mixture was poured into teflon molds, gradually heated at 80°C, 100°C, 120°C, 150°C, 180°C, 200°C and 250°C for the duration of 1 h. The modified sericite / methylphenyl-silicone resin could be obtained after the completion of the curing processes.

### 2 Characterization

Morphological features of the modified sericite / methylphenyl-silicone resin were observed by a high-resolution scanning electron microscopy (SEM) (FEI, Quanta 200F, made in the USA). Fourier-transformed infrared (FT-IR) spectrometer (Nicolet 670, made in the USA) was operated in IR region from 400 to 4000 cm^-1^ using KBr disks. X-ray diffraction (XRD) data were collected with a Shimazdu X-ray diffractometer equipped. X-ray photoelectron spectroscopy (XPS) analysis of the samples was carried out using a VG electron spectrometer (ESCALAB Mk II, made in UK). Thermogravimetric analysis (TG) was performed on a NETZSCH thermoanalyzer (STA449C, made in Germany).

## Results and discussion

### 1. Analysis of dispersity of modified sericite / methylphenyl-silicone resin

**Fig 1:**
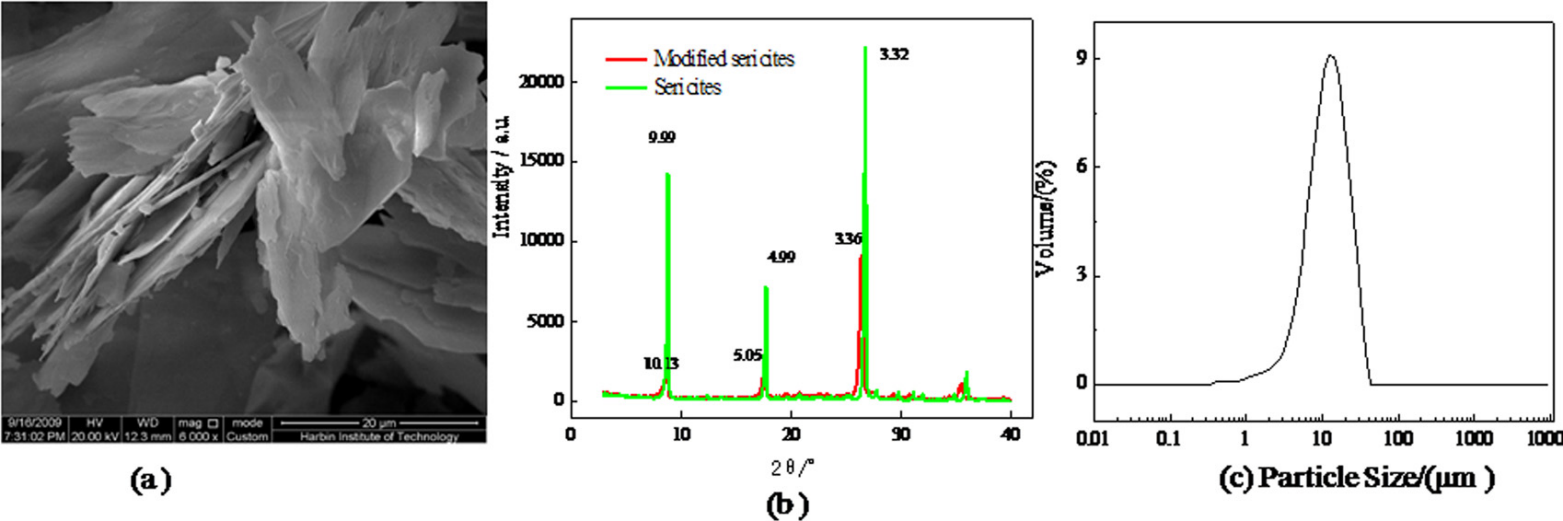
Characterization of the sericite structure: (a) SEM of the flank sericite (b) XRD patterns of the sericite and modified sericite (c) Particle of the sericite

The provided SEM images of the sericites shown in Fig 1(A), we can discover that the sericites are the layered structures with the lengths of 1–15 μm and the thicknesses of 0.8–1.5 μm. The diffractive intensities for the diffractive angles (2θ values) of 8.86, 17.78 and 26.84 for the raw sericite and 8.72, 17.56, 26.54 for modified sericite (pristine, aromatic and aliphatic) are shown in Fig 1(B). From Bragg equation, *d*-values are increased 0.14, 0.06 and 0.04, respectively. Furthermore, the size distribution of the sericite is shown in Fig 1(C). The analytical result of FT-IR spectra of the sericite and modified sericite is given in S1 Materials.

**Fig 2:**
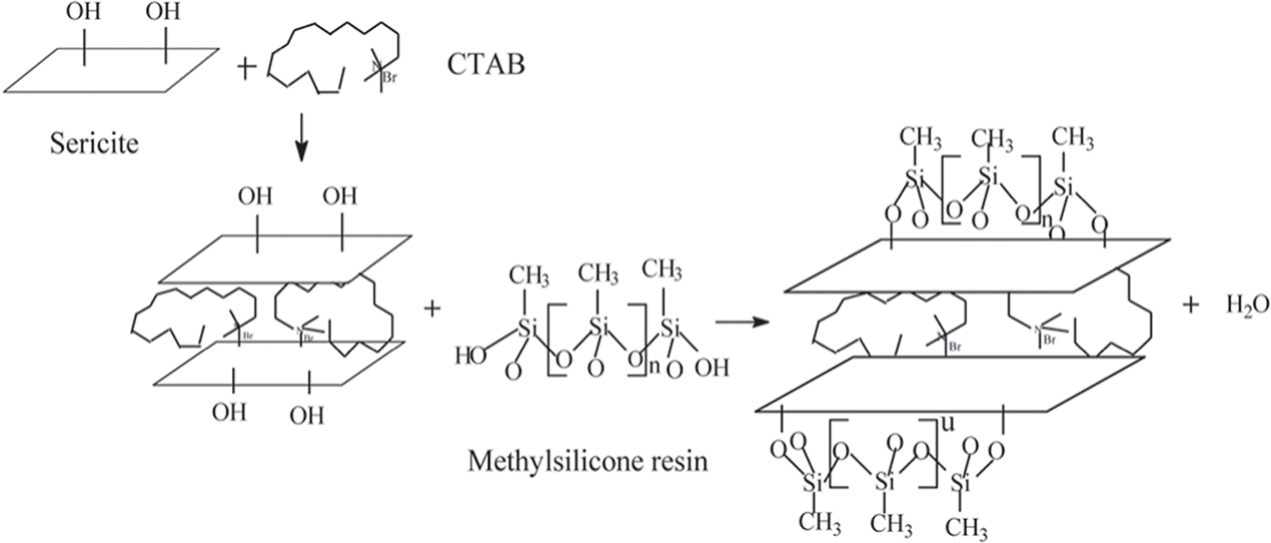
Preparation of the modified sericite /methylphenyl-silicone nanocomposite

**Fig 3:**
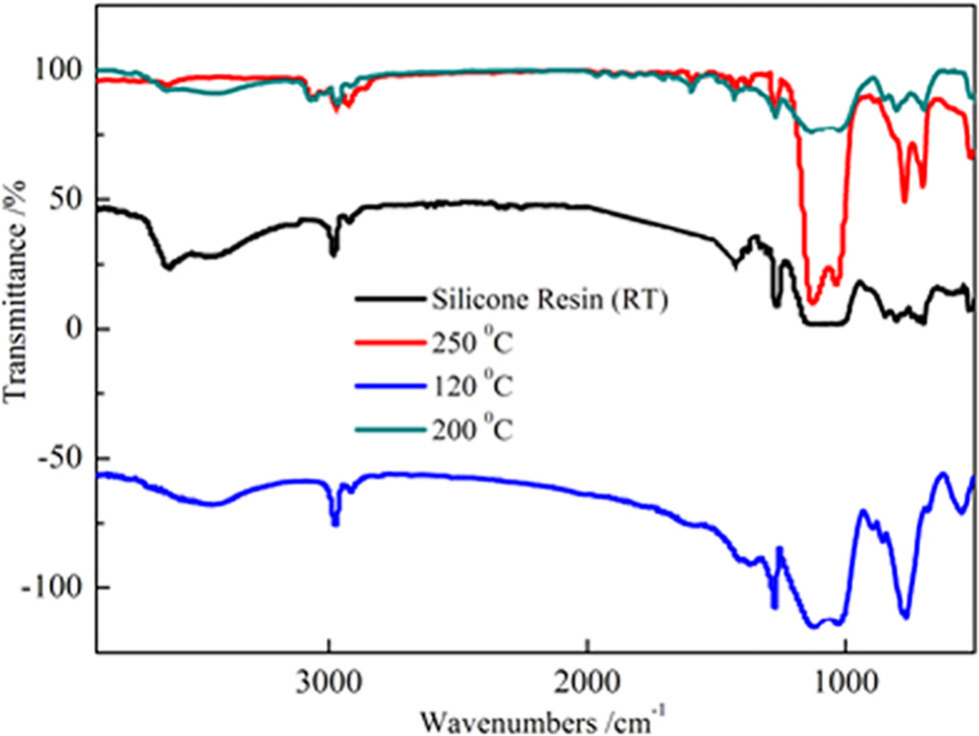
FT-IR spectra of the methylphenyl-silicone and modified methylphenyl-silicone at different temperature

The reaction process of the modified sericite / methylphenyl-silicone resin is given in Fig 2 FT-IR spectra character the molecular structure of the modified silicone resin (Fig 3). The Si-OH and Si-O-Si absorption bands of silicone resin are shown at 3620 cm^-1^ and 1160–1000 cm^-1^, respectively. The absorption peaks of the Si-OH groups at 3620 cm^-1^ are decreased, observably. The observed phenomenon suggests that the condensation reaction of the Si-OH group had taken place between the modified sericites and the methylphenyl-silicone. The other change can be observed for the peak at 1160–1000 cm^-1^. The intensity of the Si-O-Si absorption band is increased with the incorporation of modified sericites. These results further confirm that the modified sericites is indeed incorporated into the methylphenyl-silicone resin rather than as a mixture. When curing temperature increased (Room temperature, 120°C, 200°C and 250°C), the intensity of the Si-O-Si absorption peak increased and the Si-OH absorption peak decreased for the modified sericite / methylphenyl-silicone resin from Fig 3.

**Fig 4:**
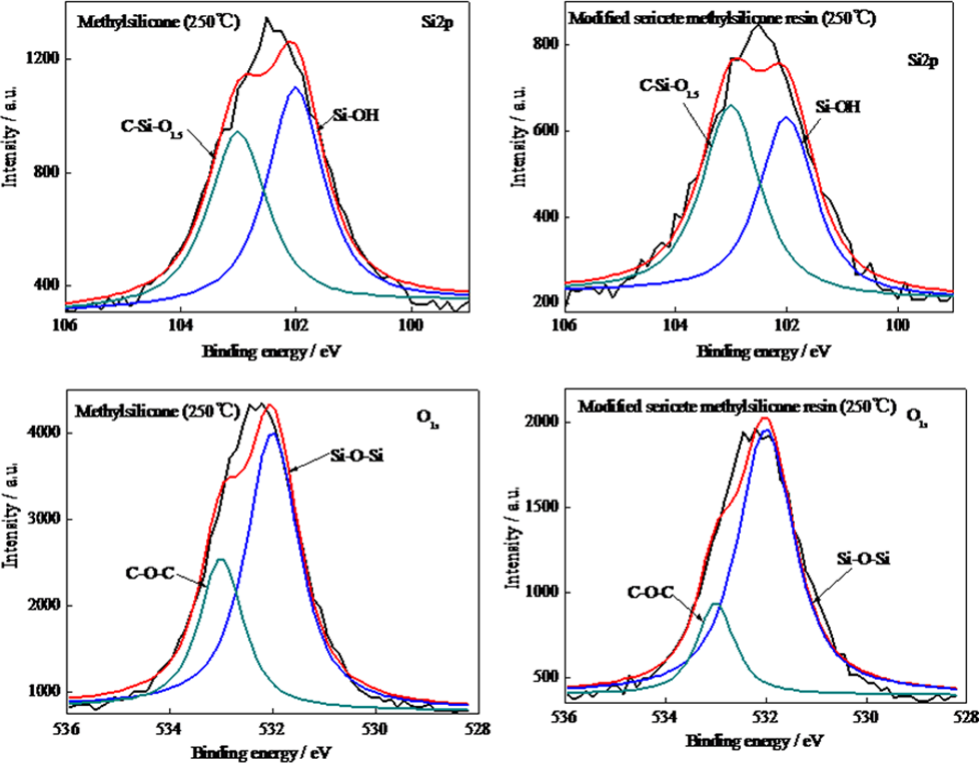
Comparison of the methylphenyl-silicone resin and modified sericite /methylphenyl-silicone: (a) Si_2p_ line (b) O_1s_ line

The high resolution XPS survey spectra showed distinct carbon, silicon and oxygen peaks, representing the major constituents of the methylphenyl-silicone resin are investigated (Fig 4). Table 1 summarizes the results of peak synthesis for the Si_2p_ and O_1s_ peaks. Fig 4(A) and 4(B) show the corresponding Si_2p_ and O_1s_ peaks for the methylphenyl-silicone and modified methylphenyl-silicone at 250°C. These peaks are centered at 103 eV which corresponds to C-Si-O_1.5_, at 102 eV which corresponds to Si-OH, in the methylphenyl-silicone chain and modified sericite. In Fig 4(A), it can be observed that the relative amount of C-Si-O_1.5_ is increased while that of Si-OH is decreased. Similarly, Fig 4(B) shows that Si-O-Si is increased while the amount of C-O-C is decresed for the modified methylphenyl-silicone. These analytical results imply that the modified sericite and methylphenyl-silicone resin has reacted. The weaker Si-OH groups are being removed and strong Si-O-Si groups are being appeared from the molecular structure of the methylphen0079l-silicone. The silica layer forms a protective barrier on the surface of modified methylphenyl-silicone, which prevents further degradation of the methylphenyl-silicone chain.

**Table 1:** Peak synthesis results of the Si_2p_ and O_1s_ peaks for the samples

Samples (250°C)	Peak Area (%)			
	O1s(eV)		Si2p(eV)	
	532	533	103	102
	Si-O-Si	C-O-C	C-Si-O1.5	Si-OH
Methylphenyl-silicone resin %	69.08	30.92	36.14	63.86
Modified methylphenyl-silicone resin %	81.46	18.54	42.08	57.92

**Fig 5:**
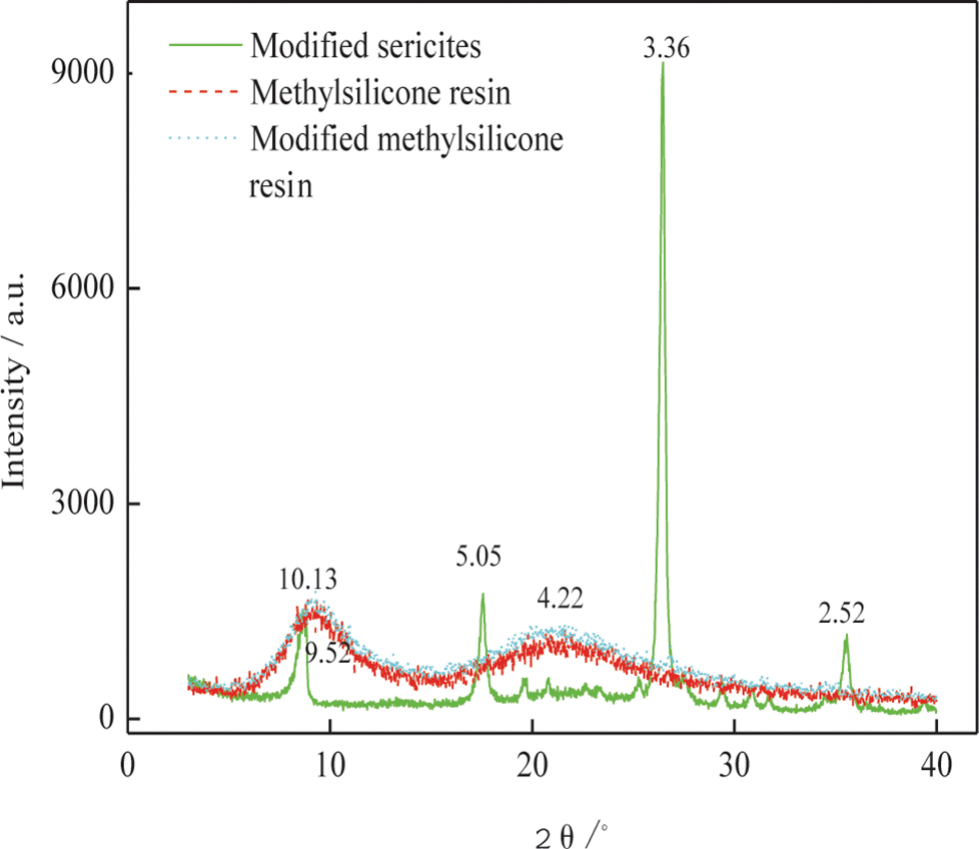
XRD patterns of the modified sericite, methylphenyl-silicone and modified methylphenyl-silicone

The collected data by XRD clearly shows that the modified sericite was exfoliated at a curing temperature of 250°C (Fig 5). After the nanocomposite is cured, all sericite particles are exfoliated and dispersed homogeneously in the modified silicone matrix. XRD of the modified sericite / silicone nanocomposite have not diffraction peak of sericite, except for polymer matrix- methylphenyl-silicone resin. This confirms modified sericite has reacted with the methylphenyl-silicone. Interestingly, no peaks corresponding to sericite or modified sericite is found, affirming the exfoliated sericites are dispersed in the silicone resin composite.

**Fig 6:**
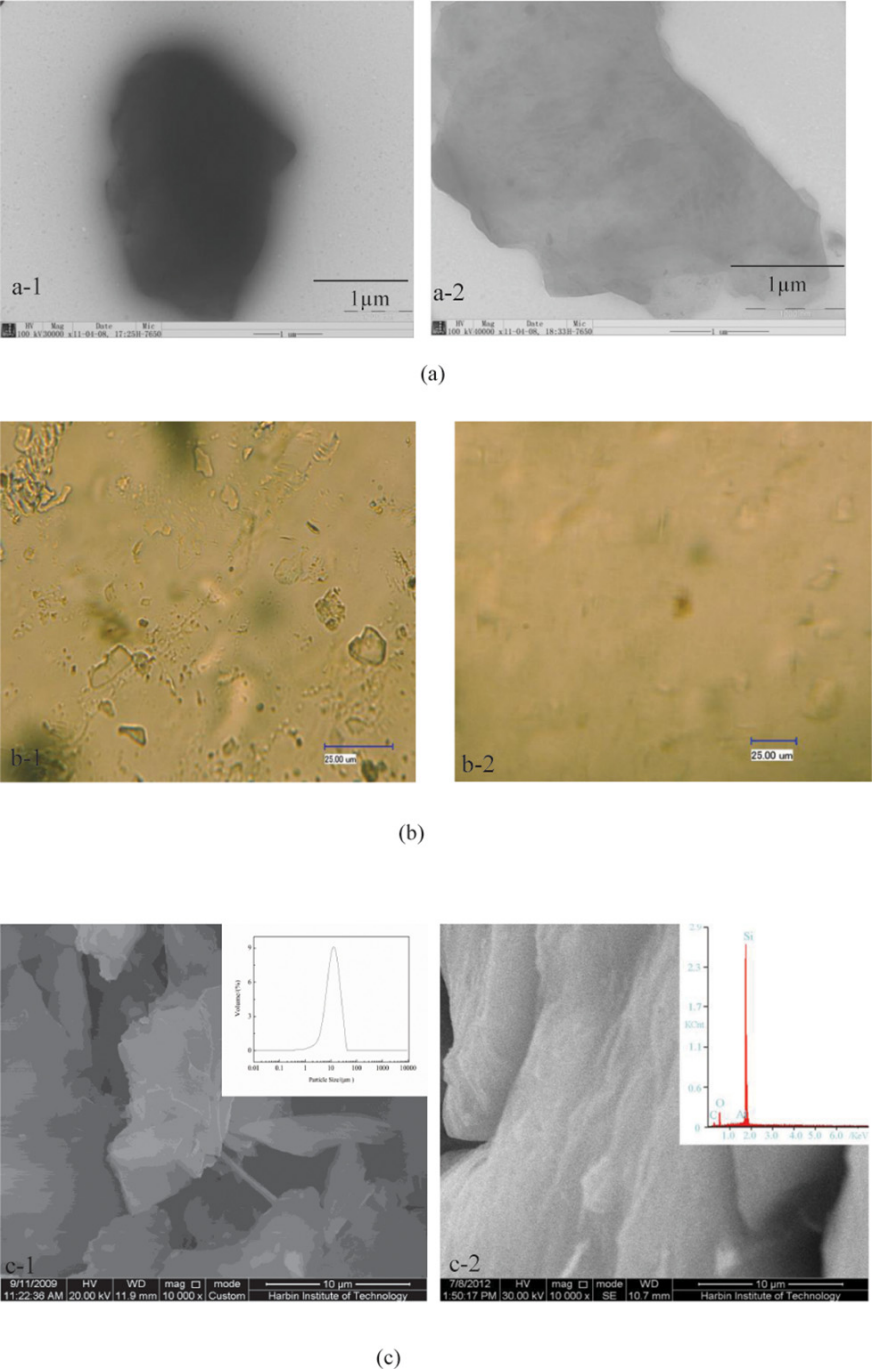
TEM images of thin sections: (a) Sericite/ methylphenyl-silicone composite cured. (b) Modified sericite/ methylphenyl-silicone nanocomposite cured. (c) EDS of the modified sericite/ methylphenyl-silicone nanocomposite

The white areas in the SEM images represent the sericite and the black areas are the methylphenyl-silicone resin (Fig 6A-1). The image shows a methylphenyl-silicone resin covers in the rather rigid sericite platelet. The compatibility of sericite/ methylphenyl-silicone resin is bad and non-uniform distribution. Fig 6(A-2) presents the homodisperse of modified sericite / methylphenyl-silicone resin nanocomposite. The surface of modified sericite is clearer and more transparent that the platelet of sericite /methylphenyl-silicone resin. In further, Laser scanning confocal microscope (LSCM) analyzed the sericite/ methylphenyl-silicone resin composite. The compatibility of modified sericite is excellent in Fig 6(B-2), unmodified sericite appear the reunite and rigid platelet. The size platelet of crude sericite is above dozens of micrometer (Fig 6C-1). The modified sericite / methylphenyl-silicone resin nanocomposite is polymerized and the large regions of pure methylphenyl-silicone matrix remain visible (Fig 6C-2). Therefore, it can be concluded that the modified sericite layers has not completely occupied the entire composite volume. It is evident that methylphenyl-silicone molecules are polymerized in the gallery region of the sericite. According to our observation, the modified sericite layers start to delaminate and exfoliate. It can be seen clearly that modified sericite is thoroughly dispersed in the methylphenyl-silicone matrix with a thickness of about several ten nanometer. The well-dispersion of the modified sericite / methylphenyl-silicone composite is also an important factor to contributing to enhance the reaction degree. In order to further analyze the microstructure of nanocomposite, the content of the main element Si, C, O and Al is studied by EDS (Fig 6C-2). This result implies that the modified sericite presents on the methylphenyl-silicone resin.

### 2. Analysis of dispersity model of modified sericite / methylphenyl-silicone resin

**Fig 7:**
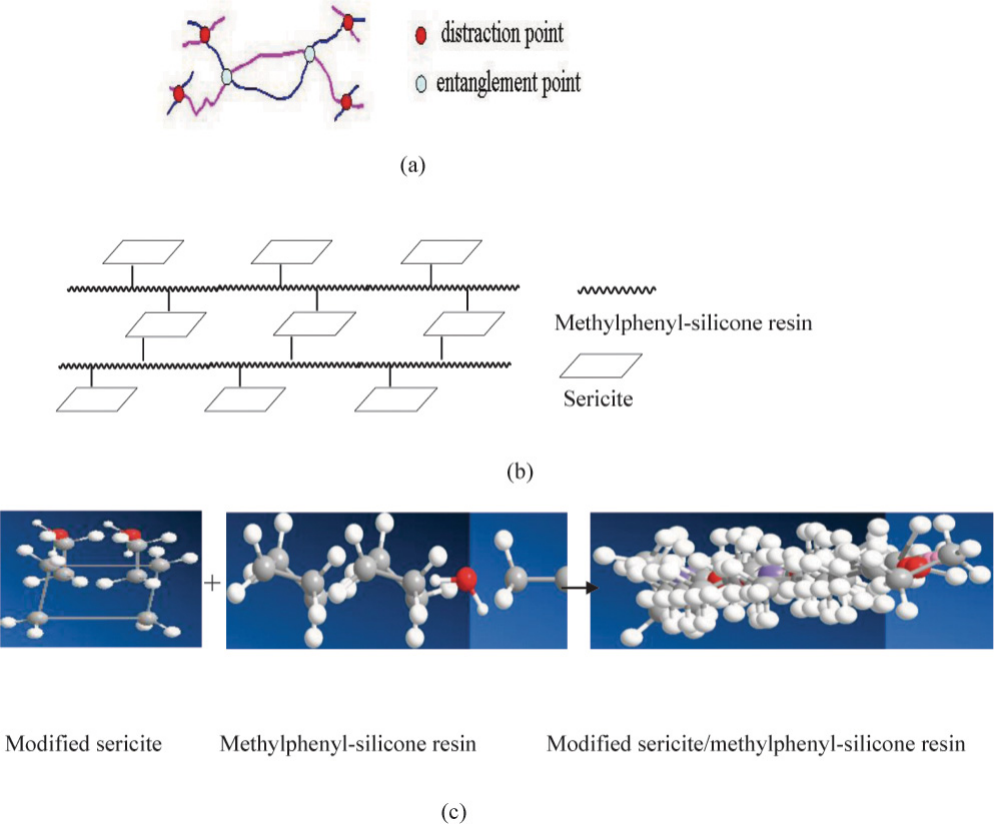
Sketch of dispersity of modified sericite / methylphenyl-silicone resin (a) Interference and entanglements in molecular chain; (b) Molecular design of dispersity model; (c) Block model of molecular chain motion

**Fig 8:**
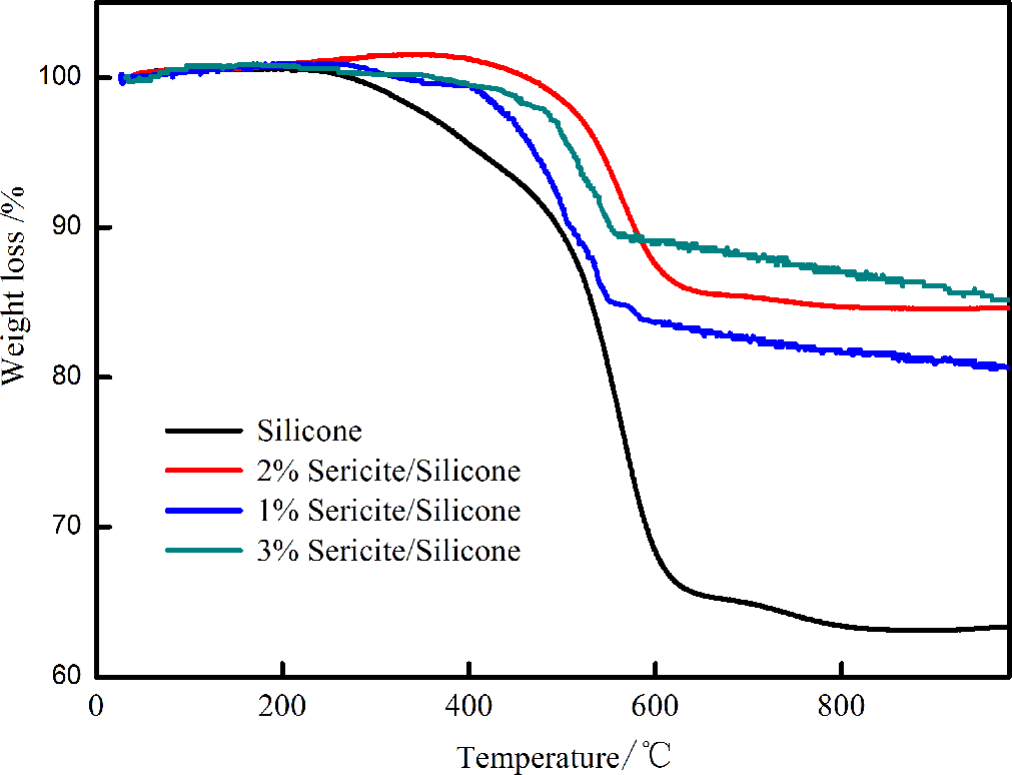
TG curves of the methylphenyl-silicone and modified methylphenyl-silicone for the different temperature

If the interference and entanglements has intermolecular or intramolecular, polymer molecular chain did not free stretching, this will affect flexibility of molecular chain. Sketch is shown in Fig 7(A). The modified sericite is dispersed in methylphenyl- silicone resin. This process will cause the physical blending and chemical reaction. From XPS analysis can discover than functional sericite was incorporated into methylphenyl-silicone by terminal hydroxyl. Morphological features of final product are shown that modified sericite uniformly dispersed in methylphenyl- silicone resin from SEM, TEM and LSCM. Dispersity models of modified sericite / methylphenyl-silicone resin are designed in Fig 7(B). The modified sericite play the part of interference and entanglement points, it hinder molecular chain motion. This process is shown in Fig 7(C). Above result has been farther verified using TG (initial silicone resin, 1wt %, 2 wt%, 3 wt % sericite/methylphenyl- silicone resin). 1wt% sericite is little, initial decomposition temperature is low. But 3 wt % influences the dispersity of the sericite/methylphenyl- silicone resin, this result causes the agglomerates of nano-particle and the poor thermal stability. So, 2 wt% is best for initial decomposition temperature. The decomposition process of methylphenyl-silicone and modified methylphenyl-silicone resin is started from the temperature values 230°C and 400°C (Fig 8), respectively. The losing weight rate for methylphenyl-silicone and modified methylphenyl-silicone is equal to 31.6% and 12.4% at 600°C, 36.9% and 15.4% at 900°C. This result implies that the molecular chain motion of methylphenyl-silicone resin with modified sericite is difficult and the decomposition temperature is higher that unmodified methylphenyl-silicone resin.

## Conclusions

Preparation of the functional sericite /methylphenyl-silicone resin composites was investigated. The modified sericite was dispersed homogeneously in methylphenyl-silicone resin matrix. Morphological features and molecular structure of product is characterizated by SEM, TEM, LSCM, XPS, XRD. From above analysis, the dispersity models of functional sericite / methylphenyl-silicone resin were designed. The study achievements have great significance in development of silicone resin composites.

## Supporting Information

S1 Materials Results analysis of sericite and modified sericite The modified process of the sericite and modified sericite is shown in the Supporting Information. FT-IR spectra are presented (**Figure A in S1 Materials**), CH_3_ and CH_2_ absorption bands is shown as 2920 cm^-1^and 2850 cm^-1^. This result shows that the hexadecyl trimethyl ammonium bromide was on the surface of the sericite or went into the interbedded structure of the sericite. After the modification, the sericite was changed from the hydrophile to the lipophilic compound. From FT-IR analytical result, it can be also deduced; crude sericite has been activated (see **S1 Materails**).
(DOC)
