# Supplementary material for: Research of the Dispersity of the Functional Sericite/Methylphenyl- Silicone Resin
Source: PLoS One. 2015 Jun 10;10(6):e0127735. doi: 10.1371/journal.pone.0127735 (PMC4465346; doi:10.1371/journal.pone.0127735)
Supplement: S1 Materials — The modified process of the sericite and modified sericite is shown in the Supporting Information. FT-IR spectra are presented (Figure A in S1 Materials), CH3 and CH2 absorption bands is shown as 2920 cm-1and 2850 cm-1. This result shows that the hexadecyl trimethyl ammonium bromide was on the surface of the sericite or went into the interbedded structure of the sericite. After the modification, the sericite was changed from the hydrophile to the lipophilic compound. From FT-IR analytical result, it can be also deduced; crude sericite has been activated (see S1 Materails). (DOC) [file pone.0127735.s001.doc]

S1 Materials

Research of the dispersity of the functional sericite/methylphenyl- silicone resin

# B. Jiang^1^*, C.C.Zhu^1^,, Y. D. Huang^1^

1. Polymer Materials and Engineering Department, School of Chemical Engineering and Technology, Harbin Institute of Technology, P.O. Box: 1254, Harbin 150001, People’s Republic of China

# * Corresponding author

Tel: 86-451-8641-4806, Fax: 86-451-8641-8270

E-mail: jiangbo5981@hit.edu.cn

**Results analysis of sericite and modified sericite**

**Figure A** shows the FT-IR spectra of the sericite and modified sericite, CH_3_ and CH_2_ absorption bands are shown as 2920 cm^-1^and 2850 cm^-1^. This result shows that the hexadecyl trimethyl ammonium bromide was on the surface of the sericite or went into the interbedded structure of the sericite. After the modification, the sericite was changed from the hydrophile to the lipophilic compound.

From FT-IR analytical result, it can be also deduced, crude sericite has been activated.

**S1Fig**. FT-IR spectravof the sericite and modified sericite
